# TCP1 increases drug resistance in acute myeloid leukemia by suppressing autophagy via activating AKT/mTOR signaling

**DOI:** 10.1038/s41419-021-04336-w

**Published:** 2021-11-08

**Authors:** Xiaofang Chen, Xianling Chen, Yiping Huang, Jia Lin, Yong Wu, Yuanzhong Chen

**Affiliations:** 1 Fujian Provincial Key Laboratory on Hematology, Fujian Institute of Hematology, Fujian Medical University Union Hospital, Fuzhou, Fujian China; 2 Department of Infectious Disease, Fujian Medical University Union Hospital, Fuzhou, Fujian China

**Keywords:** Prognostic markers, Acute myeloid leukaemia

## Abstract

T-complex protein 1 (TCP1) is one of the subunits of chaperonin-containing T complex (CCT), which is involved in protein folding, cell proliferation, apoptosis, cell cycle regulation, and drug resistance. Investigations have demonstrated that TCP1 is a factor being responsible for drug resistance in breast and ovarian cancer. However, the TCP1 role in acute myeloid leukemia (AML) remains elusive. In the present study, we discovered that the TCP1 expression was elevated in AML patients and high TCP1 expression was associated with low complete response rate along with poor overall survival. TCP1 showed higher expression in the adriamycin-resistant leukemia cell line HL60/A and K562/A, comparing to their respective parent cells HL60 and K562 cells. TCP1 inhibition suppressed drug resistance in HL60/A and K562/A cells, whereas TCP1 overexpression in HL60 cells incremented drug resistance, both in vitro and in vivo. Mechanistic investigations revealed that TCP1 inhibited autophagy and adriamycin-induced cell apoptosis, and TCP1-mediated autophagy inhibition conferred resistance to adriamycin-induced cell apoptosis. Furthermore, TCP1 interacted with AKT and mTOR to activate AKT/mTOR signaling, which negatively regulates apoptosis and autophagy. Pharmacological inhibition of AKT/mTOR signal particularly activated autophagy and resensitized TCP1-overexpressing HL60 cells to adriamycin. These findings identify a novel role of TCP1 regarding drug resistance in AML, which advise a new strategy for overcoming drug resistance in AML through targeting TCP1/AKT/mTOR signaling pathway.

## Introduction

Acute myeloid leukemia (AML) is a highly heterogeneous malignant hematological disorder that accounts for about 80% adult acute leukemia [1]. One-third of AML patients do not achieve complete remission after standard chemotherapy, and even when complete remission is achieved, ~70% of AML patients relapse within 5 years [2]. The resistance of leukemic cells to chemotherapeutic drugs is one of the main reasons for treatment failure. Hence, there is an urgent demand to elucidate the mechanisms underlying drug resistance in AML and identify new biomarkers and therapeutic targets to enhance the chemotherapy efficacy.

Chaperonin-containing T complex (CCT) is also known as the TCP1 ring complex, which is the most complex of all chaperonins with each of the two rings composed of eight paralogous subunits (here referred to as CCT1–8). Around 10% ~ 15% of cytosolic peptides are folded with the assistance of CCT to form functional proteins properly [3]. Many of the gene products deregulated in cancers, such as MYC, TP53, CCNE, KRAS, HSP90, ESR1, Cyclin E, STAT3, NOTCH, AML/ETO, and mTOR, are CCT client proteins [4–6], which advise an indispensable role for its contribution to malignant transformation. Also, there is incrementing evidence that the CCT monomers themselves may have some chaperone activities [7], which function importantly on tumorigenesis [8–11]. T-complex protein 1 (TCP1) is also known as CCT1, which is one of the eight subunits of CCT. TCP1 could promote the growth of breast cancer cells and TCP1 overexpression, which significantly correlates with reduced overall survival of breast cancer patients [10]. High TCP1 expression was discovered in cisplatin-resistant ovarian cancer cell lines and the reducing TCP1 expression incremented cisplatin-induced apoptosis and the sensitivity of the cells to cisplatin as well [11], whereas, to date, the TCP1 role in leukemia remains unreported. Our former study illustrated that the TCP1 level in highly tumorigenic HL60-G4 cells formed by repeated passages in nude mice for four generations is higher than that in the parental HL60 cells through utilizing two-dimensional difference gel electrophoresis, matrix-assisted laser desorption/ionization–tandem time-of-flight, quantitative reverse-transcriptase PCR, and western blotting analysis (unpublished data). The data indicated that TCP1 might be involved in the leukemia development. Nevertheless, the TCP1 role in AML drug resistance has not been elucidated.

The factors contributing to AML drug resistance are varied and one crucial factor is the evasion of apoptosis induced by chemotherapeutic drugs [12]. Therefore, promoting a mechanism of drug-induced cell death other than apoptosis or promoting apoptosis is one strategy to overcome drug resistance. Macroautophagy (hereafter referred to as autophagy) is a cell-based physiological process that occurs in virtually all eukaryotic cells, which has been implicated in different pathological and physiological conditions. It has been proposed that autophagy can function as a programmed cell death or promote cell death by enhancing AML cell apoptosis [13–18]. Induction of autophagy could provide an avenue to overcome drug resistance, although in some conditions, autophagy may delay cell death and autophagy induction has been shown to facilitate resistance to chemotherapeutic drugs in AML cells [19–21]. Recent study has demonstrated that CCT has a critical role in mammalian target of rapamycin (mTOR) complex assembly and signaling, which are master regulator autophagy contributing highly to drug resistance in AML [5]. However, there are still no data concerning the TCP1 role in regulating autophagy in AML.

This study aimed to investigate the TCP1 role in drug resistance concerning AML, which focus on TCP1 regulation of autophagy. A possible TCP1-mediated inhibition of autophagy via activation of AKT/mTOR signaling is proposed, which may provide a new strategy to overcome drug resistance in AML.

## Materials and methods

### Human specimens

Our team collected all samples from healthy donors and patients treated in the hematology department of Fujian Medical University Union Hospital from November 2015 to January 2018. All patients were diagnosed for AML, who received idarubicin + cytarabine induction chemotherapy. Prognosis evaluation criteria followed the National Comprehensive Cancer Network (NCCN) guidance. There were 80 peripheral blood (PB) samples from patients with AML including 66 samples from patients after the first visit and 14 samples from patients after therapy (5 samples with complete response and 9 samples with relapse), and 41 PB samples from healthy donors. There were six bone marrow samples from AML patients after the first visit and six bone marrow samples from healthy donors for bone marrow transplantation to enrich the CD34+ cells using a CD34 Microbead Kit (Miltenyi, German). Sixty-six patients included 32 male and 34 female patients aged 14–72 years (mean = 47). According to the French–American–British classification for AML classification and diagnosis, there were 1 patient with M0, 4 with M1, 19 with M2, 1 with M4, and 41 with M5 AML. Forty-one healthy controls included 20 male and 21 female donors aged 20–55 years (median = 40). This study was performed under the supervision of the Institutional Review Board of Fujian Medical University Union Hospital. All patients and healthy donors signed the informed consent.

### Cell culture

Human HL60, K562 cells, and their respective drug-resistant cells (HL60/A and K562/A) were obtained from the Institute of Hematology of Chinese Academy of Medical Sciences (Tianjin, China), which were stored at the Fujian Institution of Hematology. Our team maintained the cells in RPMI-1640 medium (Hyclone) supplemented with 10% fetal bovine serum (FBS; Gibco, Billings, MT, USA). We cultured HL60/A cells in 1 µg/ml doxorubicin (ADM) to maintain resistance. Our lab cultured K562/A cells in 5 µg/ml ADM to maintain resistance. ADM was eliminated 1 month prior to testing. Next, 293T cell line was cultured in Dulbecco’s modified Eagle’s medium (Hyclone) supplemented with 10% FBS. We examined cellular identities by their karyotypes and morphologies, and all cells were verified to be pollution free of mycoplasma.

### Lentiviral vectors and transduction

We transfected TCP1-specific targeting short hairpin RNA (shRNA) vector or empty vector with expression and packaging vectors into 293T producer cells employing Lipofectamine™ 2000 (Invitrogen, USA), following the protocol. Lentivirus with TCP1-shRNA vector and the control vector were transfected into HL60/A and K562/A cells, respectively. Our team purchased ATG7-small interfering RNA (siRNA) lentiviral vector and its corresponding control vector from Genechem (Shanghai, China), which were transduced into HL60/A-shTCP1 cells. The TCP1 overexpression lentiviral vector and its corresponding scrambled control vector were obtained from Vigene Bioscience (Shangdong, China), which were transduced into HL60 cells (multiplicity of infection = 150) separately. Our team selected cells that transduced with puromycin (2 µg/mL).

The sequences of the RNA were as follows:

*TCP1-shRNA/F: 5*′*-CCGGGGTGTACAGGTGGTTCATTATTCAAGAGATAATGACCACCTGTACACC-3*′*; TCP1-shRNA/R: 5*′*-AATTCAAAAAAGGTGTACAGGTGGTCA TT AT CT CTTGAATAATGACCACCTGTACACC-3*′*; and ATG7-siRNA: 5*′*-GCCTGCT GAGGAGCTCTCCAT-3*′*.*

### Quantitative real-time reverse transcription PCR

We determined relative *TCP1* mRNA expression by real-time quantitative PCR and 2^−ΔCt^ methods, comparing TCP1 expression relative to glyceraldehyde 3-phosphate dehydrogenase (*GAPDH*). Patients with *TCP1* expression values above the median were defined to have high *TCP1* expression (*TCP1*^high^), whereas the rest of the patients were considered to have low *TCP1* expression (*TCP1*^low^).

### PCR array screening

Expression of genes involved in drug resistance (such as autophagy, apoptosis, DNA damage response, and metabolism) was quantified through RT2 Profiler PCR array following standard protocol (Wcgene Biotech, Shanghai, China). We analyzed data utilizing Wcgene Biotech software. One hundred and sixty genes were tested. Gene expression was regarded to be changed if the fold regulation of the target genes was significantly different from the control group (Student’s *t*-test, *p* < 0.05, *n* = 3).

### Western blot analysis

Our team performed western blot analysis employing antibodies to LC3A/B (#12741), total AKT (#4691), phosphorylated AKT (#4060), phosphorylated-mTOR (#2972), total mTOR (#2971), phosphatidylinositol 3-kinase (PI3K) (#4249), cleaved caspase-3 (#9662), and cleaved poly (ADP-ribose) polymerase (PARP) (#94885) from Cell Signaling Technology (Danvers, MA, USA), and p62 (ab109012), ATG7 (ab52472), TCP1 alpha (ab109126), and GAPDH (ab181602) from Abcam (Cambridge, UK), following the protocols.

### Transmission electron microscopy

Our team centrifuged cells that treated to remove the supernatant, which were fixed with fixation solution (4% paraformaldehyde, 0.25% glutaraldehyde in 0.1 M phosphate buffer pH 7.4) at 4 °C over the night. The cells were treated following the protocol of Fujian Medical University (Fuzhou, China). We observed ultrathin sections under transmission electron microscope (Quanta 450, FEI, USA).

### Immunofluorescence staining and confocal fluorescence microscopy

Cells were centrifuged, smeared on slides, and fixed with 100% ice-cold methanol for 20 min at −20 °C. After being washed with phosphate-buffered saline (PBS), we blocked the slides with closure buffer (1× PBS/5% normal serum/0.3% Triton X-100) for 1 h. We then incubated cells with LC3A/B primary antibody (diluted 1 : 200) at 4 °C overnight and with Alexa Fluor 488-conjugated goat anti-rabbit IgG secondary antibody (ab150077; Abcam) for 1 h at 25 °C. Our lab stained nuclei with 4′,6-diamidino-2-phenylindole dye for 5 min, which were blocked with Antifade Reagent. Finally, we acquired and analyzed images utilizing confocal fluorescence microscope (TCS SP8, Leica, Germany). Immunofluorescence staining of TCP1 and LC3A/B in tumors was performed following the manufacturer’s protocol (Servicebio, Wuhan, China).

### Autophagy detection by autophagy fluorescence imaging

According to the protocol of Cell Meter Autophagy Fluorescence Imaging Kit (23001; AAT Bioquest, Sunnyvale, CA, USA), we added 250 µL Autophagy Super Blue working solution to cells. Afterwards, we incubated the cells at 37 °C for 30 min, which were protected from light in a 5% CO_2_ environment. After washing three times with wash buffer, we detected autophagy under fluorescence microscope and acquired the photos.

### Co-immunoprecipitation

We collected and lysed cells with radioimmunoprecipitation assay (RIPA) buffer containing protease inhibitors, phosphatase inhibitors, and phenylmethylsulfonyl fluoride on ice. Our lab precleared cell lysates with 1.0 µg control IgG and 20 µL resuspended Protein A/G PLUS-Agarose (sc-2003; Santa Cruz Biotechnology) by incubation for 1 h at 4 °C on a shaker. Next, the cleared supernatant (500 µL each) was incubated for 1 h with anti-TCP1 or homotype control IgG, which were mixed with 20 µL resuspended Protein A/G PLUS-Agarose and incubated overnight at 4 °C on a shaker. On the following day, we washed samples four times with RIPA buffer. Immobilized protein complexes were denatured with 2× SDS sample buffer at 100 °C for 5 min, for subsequent western blotting.

### Cell viability assays

We seeded 1 × 10^4^ cells in 96-well plates, which were cultured with RPMI-1640 complete medium with the addition of various drug concentrations. Cell viability was assessed through the Cell Counting Kit-8 (Dojindo, Japan) following manufacturer’s instructions. The concentration at which each drug produced 50% inhibition of growth (IC_50_) was estimated by relative survival curve.

### Flow-cytometric apoptosis assay

The Apoptosis Detection Kit (Biolegend, USA) was utilized to detect apoptosis following the manufacturer’s instructions, which was analyzed by flow cytometry (BD FACScan flow cytometer, BD Biosciences, USA).

### AML xenograft animal model

Ethics Committee for Animal Studies in Fujian University reviewed and approved animal studies (number FJMUIACUC 2018-103). Four-week-old male nude mice were purchased from Shanghai Slac Laboratory Animal Co., Ltd, Shanghai, China. We maintained all mice in specific pathogen-free conditions in the animal facility at Fujian Medical University. The nude mice were subjected to 100 mg/kg/d cyclophosphamide by intraperitoneal injection for two consecutive days. Cells (2 × 10^7^) were subcutaneously injected into the right flanks of the nude mice. When the volume of the xenografts reached 50 mm^3^, mice were randomly divided into four groups: HL60/NC and HL60/TCP1 with solvent or ADM treatment (3 mg/kg/d via intraperitoneal injection for 3 days, 5 mice per group). Tumor size was captured every 3 days with caliper (tumor volume = [shortest diameter^2^ × longest diameter]/2). After 1 month, we killed the mice and the tumors were weighed and prepared for next-step analysis.

### Statistical analysis

GraphPad Prism 7 and SPSS 23.0 (IBM SPSS Statistics 23) software were utilized for statistical analysis. Then, *χ*^2^- or Fisher’s exact tests were performed to compare incidences. The Kaplan–Meier method was employed to estimate survival probabilities and the log-rank test was employed for univariate comparisons. Matched-pairs *t*-test was utilized to compare two groups of samples. Each cellular experiment was repeated for at least three times. Significant differences between groups were analyzed by Student’s *t*-test. The results are expressed as mean ± SEM. *P* < 0.05 represented a significant difference (*P < 0.05, **P < 0.01, ***P < 0.001).

## Results

### TCP1 upregulation is associated with poor prognosis of AML patients

**Fig. 1:**
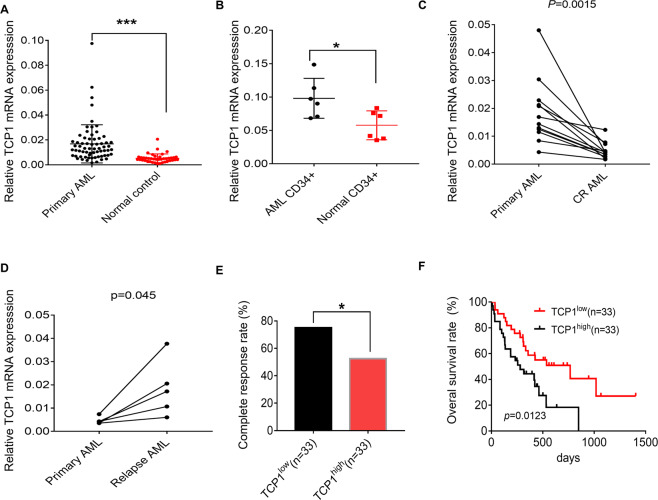
*TCP1* upregulation is associated with poor prognosis in AML patients. **A** *TCP1* mRNA was significantly upregulated in PB cells from primary AML patients (*n* = 66) compared with normal control. **B** TCP1 mRNA was increased in CD34 + BM cells from AML patient vs. healthy control donors. **C** *TCP1* mRNA was significantly upregulated in PB cells in relapsed AML patients. **D** *TCP1* mRNA was decreased in PB cells of AML patients in complete remission. **E** Patients from the *TCP1*^low^ group presented a significantly higher rate of complete response (CR) rates than patients from the *TCP1*^high^ group (*P* = 0.041). **F** Patients from the *TCP1*^low^ group presented significantly longer overall survival (OS) than patients from the *TCP1*^high^ group (*P* = 0.0123) (*TCP1* expression was normalized to *GAPDH* expression). *P < 0.05, **P < 0.01, ***P < 0.001.

To investigate the potential clinical significance of TCP1 in leukemia, we examined the relative expression of *TCP1* mRNA in PB mononuclear cells from 66 newly diagnosed leukemia patients and 41 healthy donors. *TCP1* mRNA expression was significantly higher in AML patients in comparison to those in healthy controls (Fig. 1A). *TCP1* mRNA overexpression was also confirmed in human AML CD34+ cells in comparison with that in normal CD34+ cells (Fig. 1B). Meanwhile, *TCP1* mRNA expression in AML patient was upregulated when the patient relapsed (Fig. 1C) and it was reduced when the patient was in complete remission (Fig. 1D). Those patients in the *TCP1*^low^ groups had a significantly higher rate of complete response (*P* = 0.041) and a significantly longer overall survival (*P* = 0.0123) than patients in the *TCP1*^high^ group (Fig. 1E, F). These data indicate that *TCP1* is a risk factor for AML and high *TCP1* expression is associated with poor prognosis of AML patients.

### TCP1 expression is negatively correlated with drug sensitivity in AML cells

**Fig. 2:**
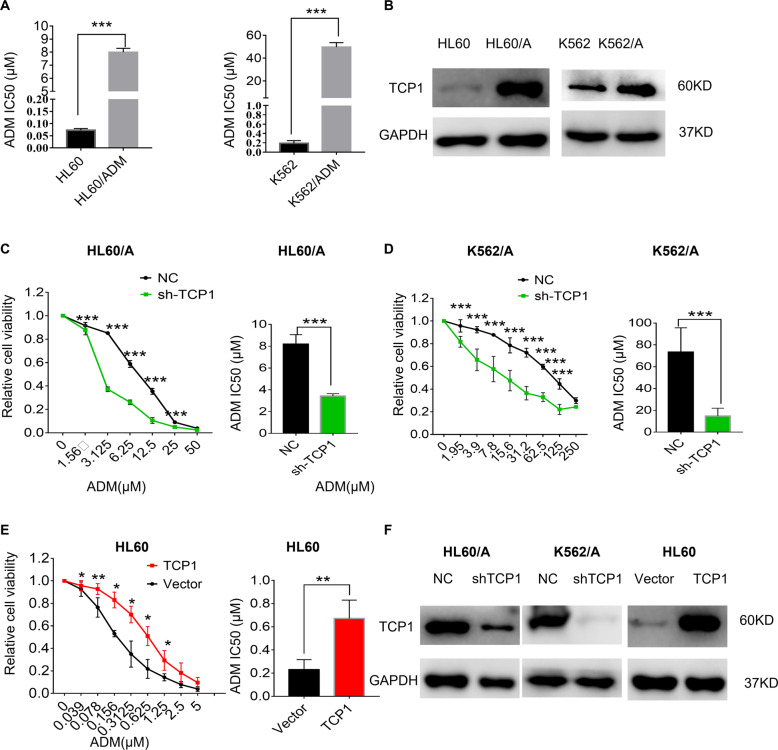
TCP1 contributes to the drug resistance of AML cells to ADM. The viability of HL60/A, K562/A cells, and their respective parent cells under the indicated dose of ADM for 48 h was detected by the Cell Counting Kit-8 (CCK-8) assay and the IC50 of ADM was calculated by spss (**A**). **B** The level of TCP1 was determined by western blotting. HL60/A and K562/A cells transfected with TCP1-specific shRNA lentivirus vector and HL60 cells transfected with a TCP1-overexpressing lentivirus vector; the effect of expression of TCP1 on the viability was measured via the CCK-8 assay after ADM treatment for 48 h and the ADM IC50 was calculated by SPSS (**C**–**E**), the efficiency of the knockdown or overexpression was confirmed by western blottin (**F**). Data are expressed as mean ± SEM; *P < 0.05, **P < 0.01, ***P < 0.001.

To investigate the TCP1 role in drug sensitivity for AML cells, we first evaluated the viability of HL60, K562 cells, and their respective multidrug-resistant cells (HL60/A and K562/A) cultured with different ADM concentrations. Results from the cell viability assay validated that each of the drug-resistant clones had marked resistance to ADM in comparison to their parental cells (Fig. 2A). We detected the TCP1 expression in cells through western blotting. The TCP1 levels in HL60/A and K562/A cells were higher than those in their parental cells (Fig. 2B). To explore whether the cellular level of TCP1 is related to the resistance of AML cells to ADM, we transiently transfected HL60/A and K562/A cells, which have a relatively higher TCP1 expression, with TCP1-specific shRNA lentivirus vector to knock down TCP1 expression. We transfected HL60 cells, which have a relatively lower TCP1 expression, with a TCP1 overexpression lentivirus vector to overexpress TCP1. The cell viability assay data advised that knocking down TCP1 in HL60/A and K562/A cells led to enhanced ADM-induced suppression of cell viability and decreased IC50 of ADM (Fig. 2C, D). In contrast, TCP1 overexpression in HL60 cells partially reversed the ADM-induced inhibition of cell viability and increased the IC50 of ADM (Fig. 2E). The efficiency of the knockdown or overexpression was confirmed by western blotting (Fig. 2F). The results indicated that TCP1 contributed to protection of AML cells against ADM treatment.

### TCP1 inhibits autophagy and drug-induced apoptosis in AML cells

Many mechanisms of drug resistance were established [12]. To better understand which mechanisms of drug resistance are mainly due to TCP1, we performed a drug-resistant PCR array analysis. This analysis revealed that the TCP1 knockdown obviously upregulated many autophagy- or apoptosis-related genes (Table 1), indicating that TCP1 may be involved in autophagy and apoptosis.

**Table 1:** Autophagy- and apoptosis-related genes display increased fold changes in HL60/A-shTCP1 cells compared with HL60/A-NC cells.

Gene description	Gene symbol	fold change	*P*-value
Autophagy-related 12	*ATG-12*	2.001511721	*
Autophagy-related 16 like 1	*ATG-16L1*	2.419457513	*
Autophagy-related 3	*ATG3*	2.771253725	*
Autophagy-related 4B cysteine peptidase	*ATG4B*	2.303244403	*
Autophagy-related 4D cysteine peptidase	*ATG4D*	2.052146411	*
Autophagy-related 5	*ATG 5*	2.704973958	*
Autophagy-related 7	*ATG 7*	0.005279354	**
Autophagy-related 9A	*ATG 9A*	3.502362156	*
Autophagy-related 9B	*ATG 9B*	2.762346476	*
ATM serine/threonine kinase	*ATM*	2.092491868	*
BCL2 associate agonist of cell death	*BAD*	1.758527873	*
BCL2 antagonist/killer 1	*BAK1*	2.60204395	*
Beclin 1	*BECN1*	2.234079666	*
BH3-interacting domain death agonist	*BID*	1.616271877	*
Caspase-3	*CASPS*	1.695871192	*
Cyclin D1	*CCND1*	2.059370653	*
DNA damage-regulated autophagy modulator	*DRAM1*	2.243884459	*
Histone deacetylase 1	*HDAC1*	2.187058231	*
Lysosomal-associated membrane protein 1	*LAMP1*	3.133350175	*
Mitogen-activated protein kinase 14	*MAPK 14*	2.592001772	*
Phosphatase and tensin homolog	*PTEN*	1.502452326	*
Retinoid X receptor beta	*RXRB*	2.042668755	*
Superoxide dismutase	*SOD1*	2.486531674	*
T-complex 1	*TCP1*	0.210511585	*
Tumor necrosis factor	*TNF*	1.783959997	*
Unc-51-like autophagy activating kinase 2	*ULK2*	6.94632296	*

*P < 0.001, **P < 0.01*.*

**Fig. 3:**
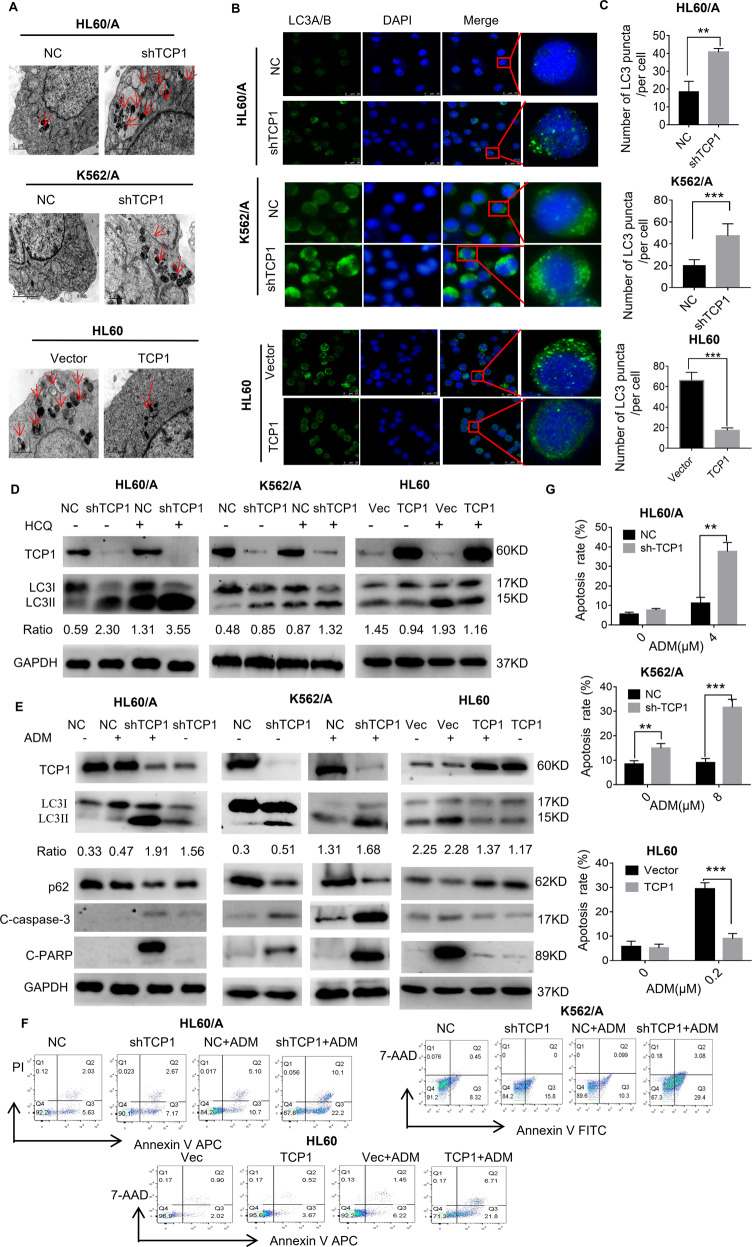
TCP1 inhibits autophagy and apoptosis in AML cells. **A** Transmission electron microscopy images showing autophagosome formation in the cell lines; the arrows indicate autophagic vesicles (scale bar = 1 μm). **B** Cells were stained with anti-LC3A/B antibody and DAPI to detect the endogenous LC3 puncta and nuclei, respectively, by immunofluorescence using a confocal microscope. Scale bar = 25 μm. **C** The quantitative analyses of the number of fluorescent puncta are shown. **D** Western blot analysis of LC3-II, LC3-I, and TCP1 expression levels in the presence or absence of 10 μM HCQ in cells. Cells were treated with different doses of ADM for 48 h. **E** The autophagy- and apoptosis-related protein were detected by western blot assay. **F** Then cell apoptosis was measured by an Annexin V dual-staining assay followed by flow cytometry and **G** the percentage of early and late-apoptotic cells were quantified. Data are expressed as mean ± SEM; *P < 0.05, **P < 0.01, ***P < 0.001.

Autophagic vacuoles detected by transmission electron microscopy, endogenous LC3 puncta detected by immunofluorescence staining, and the increasing ratio of cellular LC3-II to LC3-I detected by western blotting are classical markers of autophagosome formation [22]. To confirm the potential link between TCP1 and autophagy in AML, we analyzed autophagic vacuoles by transmission electron microscopy, endogenous LC3 puncta by immunofluorescence staining, and the level of LC3 by western blotting in cells in which TCP1 was silenced or overexpressed. The data showcased that the TCP1 knockdown in HL60/A and K562/A cells increased autophagic vacuoles, the number of endogenous LC3 puncta, the LC3-II level, and the LC3-II/LC3-I ratio (Fig. 3A–D). The increased LC3-II may be due to either autophagy induction or inhibition of autophagic flux [23]. To further confirm TCP1 regulation of autophagic flux, we utilized hydroxychloroquine (HCQ), which was an inhibitor of autophagy and lysosome fusion. Knocking down TCP1 in the presence of HCQ resulted in higher LC3-II levels and higher LC3-II/LC3-I ratio comparing with knocking down TCP1 alone or HCQ treatment alone, which indicate that the increase in LC3-II is due to increased production rather than decreased recycling of LC3-II (Fig. 3D). The similar results were found in TCP1 knocking down of HL60 cells (Supplementary Fig. 1A, B). Consistently, TCP1 overexpression in HL60 cells resulted in the reverse phenomenon (Fig. 3A–D). In addition, western blot analysis also showcased that combined ADM treatment with TCP1 knockdown further incremented the LC3-II level and LC3-II/LC3-I ratio comparing with TCP1 knockdown alone or ADM treatment alone, whereas TCP1 overexpression combined with ADM treatment resulted in a lower LC3-II/LC3-I ratio comparing with cells treated with ADM alone (Fig. 3E). Collectively, all the results indicate that TCP1 inhibits autophagy and ADM induced autophagy in AML cells.

To explore the TCP1 effect on cell apoptosis, we performed apoptosis detection by flow cytometry, which showed that TCP1 knockdown in HL60/A and K562/A cells significantly increased ADM-induced apoptosis, whereas the TCP1 overexpression in HL60 cells significantly inhibited ADM-induced apoptosis (Fig. 3F, G). The results were further validated by western blotting, which showcased that ADM significantly incremented the cleaved caspase-3 and cleaved PARP levels in HL60/A-shTCP1 and K562/A-shTCP1 cells in comparison with control cells, whereas TCP1 overexpression in HL60 cells inhibited the ADM-induced increment in cleaved caspase-3 and cleaved PARP levels (Fig. 3E).

### TCP1 inhibited autophagy contributes to drug resistance of AML cells

**Fig. 4:**
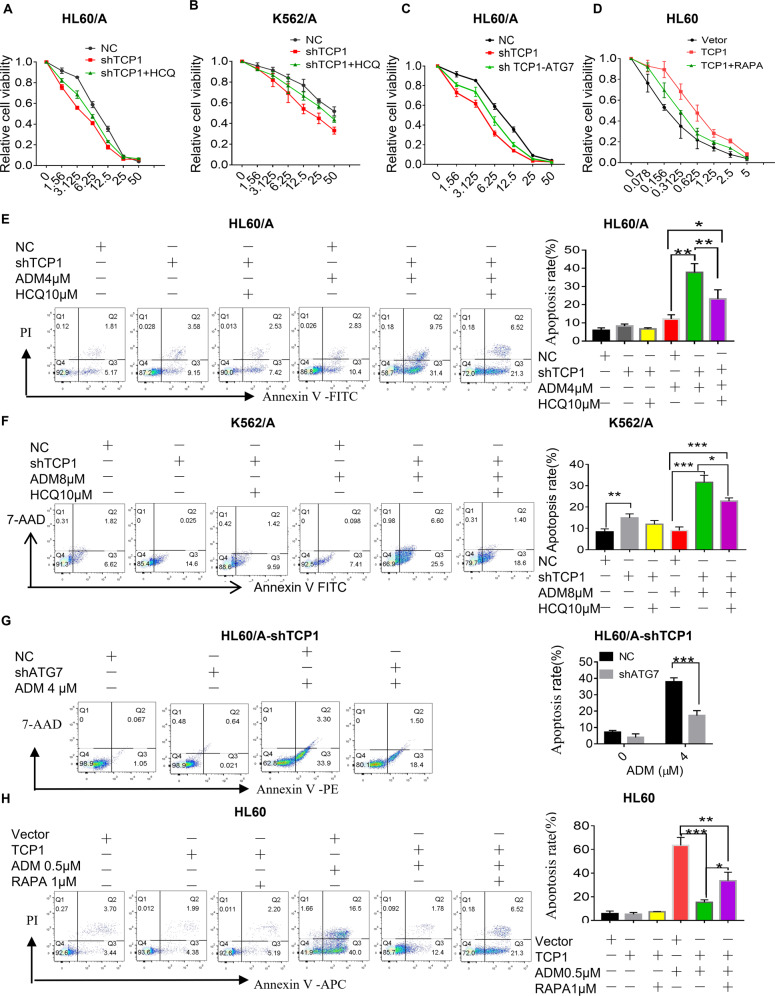
Inhibition of autophagy by TCP1 contributes to the drug resistance of AML cells. **A**, **B** HL60/A-shTCP1 and K562/A-shTCP1 cells were treated with HCQ (0 and 10 μM) for 2 h. **C** HL60/A-shTCP1 cells were transfected with ATG7 shRNA or negative control for 72 h. **D** HL60/TCP1 cells were treated with rapamycin (0 μM, 500 nM, 1 μM) for 2 h, then in combination with the indicated dose of ADM for 48 h. The cell viability was measured by the CCK-8 assay. **E**, **F** HL60/A-shTCP1 cells, K562/A-shTCP1, and their respective control cells were treated with or without 4 μM ADM for 48 h. HL60/A-shTCP1 and K562/A-shTCP1 cells were treated with or without 4 μM ADM after incubation with 10 μM HCQ for 2 h. Then, apoptotic cells were assessed using flow cytometry and the percentage of early and late-apoptotic cells were quantifed. **G** HL60/ADM-shTCP1 cells were transfected with ATG7 shRNA or negative control (NC) for 72 h and then treated with ADM (4 μM) for 48 h. Then, apoptotic cells were assessed using flow cytometry and the percentage of early and late-apoptotic cells were quantifed. **H** HL60/TCP1 cells and HL60/Vector cells treated with or without 0.5 μM ADM for 48 h, and HL60/TCP1 cells were treated with or without 0.5 μM ADM after incubation with rapamycin (RAPA, 1 μM) for 2 h. Then, apoptotic cells were assessed using flow cytometry and the percentage of early and late-apoptotic cells were quantifed. Data are expressed as mean ± SEM; *P < 0.05, **P < 0.01, ***P < 0.001.

Rapamycin (RAPA, an agonist of autophagy) could inhibit mTOR signaling and activate autophagy (Supplementary Fig. 2A, B), which promoted ADM-induced apoptosis in HL60/A cells and K562/A cells (Supplementary Fig. 2B, C). These results revealed that autophagy might contribute to the chemosensitivity of ADM in HL60/A and K562/A cells. We evaluated whether TCP1-mediated inhibition of autophagy contributed to the development of drug resistance in AML cells. The results of cell viability assays showed that TCP1 knocking down in HL60/A and K562/A cells significantly increased ADM-induced inhibition of cell viability and this effect was attenuated by HCQ treatment or ATG7 knockdown, which inhibits autophagy (Fig. 4A–C), whereas the TCP1 overexpression in HL60 cells significantly decreased ADM-induced inhibition of cell viability and this effect was partially reversed by RAPA (Fig. 4D). Apoptosis detection using flow cytometry illustrated that knocking down TCP1 in HL60/A and K562/A cells significantly increased ADM-induced apoptosis and this effect was attenuated by HCQ treatment or ATG7 knockdown (Fig. 4E–G). In contrast, the TCP1 overexpression in HL60 cells significantly decreased ADM-induced apoptosis and this effect was reversed partially by RAPA (Fig. 4H). Furthermore, western blot analysis further confirmed the above results of flow cytometry by detecting the levels of cleaved caspase-3, cleaved PARP, p62, and LC3-II/LC3-I (Supplementary Fig. 3A–D).

### TCP1 inhibits autophagy and drug-induced apoptosis by regulating the AKT/mTOR signaling cascade

**Fig. 5:**
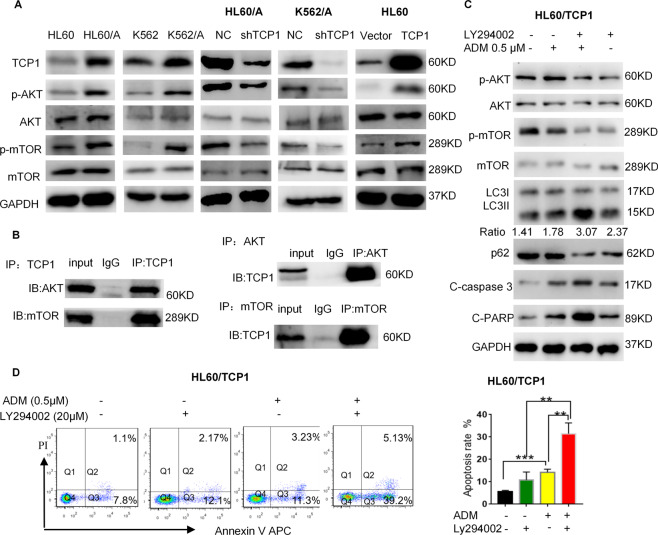
TCP1 inhibits autophagy through regulating the AKT/mTOR signaling cascade. **A** The expressions of TCP1, p-AKT, AKT, p-mTOR, mTOR, and GAPDH were detected by western blotting in cells. **B** Co-immunoprecipitation of TCP1 and AKT or mTOR in HL60/A cells. HL60/ TCP1 cells were pretreated with LY294002 (20 μM) or control for 2 h and then treated with ADM (0.5 μM) or vehicle for 48 h, then the expressions of TCP1, AKT/mTOR signaling-related factors, and autophagy- and apoptosis-related protein were detected by western blotting (**C**) and apoptotic cells were assessed using flow cytometry, and the percentage of early- and late-apoptotic cells were quantified. **D** Data are expressed as mean ± SEM; *P < 0.05, **P < 0.01, ***P < 0.001.

We analyzed the mechanisms relating to the TCP1 role in the autophagic process. The mTOR signaling pathway is crucial for autophagy induction [24, 25]. AKT/mTOR activation is associated with poor prognosis in AML, which strongly contributes to drug resistance [26–28]. We compared AKT/mTOR activation between HL60/A, K562/A, and their respective parental cells. The phosphorylation levels of AKT and mTOR in HL60/A cells and K562/A were higher than those in their respective parental cells, which is consistent with the TCP1 expression levels in the two cell lines (Fig. 5A). Knocking down TCP1 of HL60/A and K562/A cell decreased the protein levels of phosphorylated-AKT and phosphorylated-mTOR, whereas TCP1 overexpression significantly increased the protein levels of phosphorylated-AKT and phosphorylated-mTOR (Fig. 5A). Immunoprecipitation experiments examined the potential associations between TCP1, AKT, and mTOR in HL60/A cells. The data showcased that immune-precipitated TCP1 co-precipitated AKT and mTOR, and immune-precipitated AKT and mTOR co-precipitated TCP1 (Fig. 5B). Thus, TCP1 can physically interact with AKT and mTOR to regulate AKT/mTOR signaling. We determined whether TCP1 regulated autophagy and apoptosis through AKT/mTOR signal. LY294002 (S1105; Selleck) was a PI3K/AKT/mTOR inhibitor, which inhibited the activation of AKT/mTOR signaling in HL60/TCP1 cells, increased LC3-II conversion, and decreased p62 levels (Fig. 5C). Moreover, combined treatment of HL60/TCP1 cells with LY294002 and ADM inhibited AKT/mTOR signal, which was activated by TCP1 overexpression and promoted ADM-induced cell autophagy and apoptosis (Fig. 5C, D). Similar results were obtained using RAPA combined with ADM (Fig. 4H and Supplementary Fig. 3D). In summary, our results indicated that TCP1 inhibited autophagy and ADM-induced apoptosis by activating the AKT/mTOR pathway.

### TCP1 overexpression in HL60 cells attenuates the antitumor activity of ADM in vivo

**Fig. 6:**
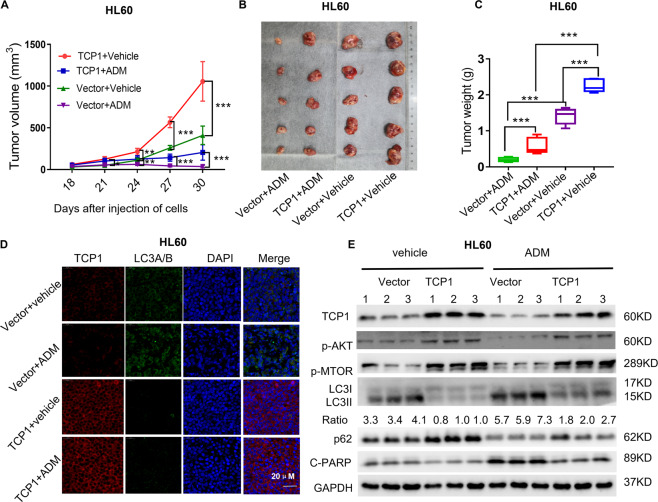
Overexpression of TCP1 in HL60 cells promotes ADM resistance in vivo. HL60/Vect or HL60/TCP1 cells were subcutaneously injected into male nude mice, and the mice were treated with ADM (3 mg/kg/day) or vehicle for 3 days intraperitoneally. The mice were killed after 30 days. **A** Tumor volumes in the four groups were measured with Vernier calipers every 3 days. **B**, **C** After the mice were killed, the tumors were excised and weighed (**C**), and representative images of tumor xenografts are shown (**B**). **D** The expression of TCP1 and LC3 was detected by immunofluorescence in the tumors from the four groups. **E** The expression levels of TCP1, p-AKT, p-mTOR, LC3-II/I, p62, and cleaved PARP were determined by western blotting in three representative mice from the different groups. The results represent the mean ± SEM; *P < 0.05, **P < 0.01, ***P < 0.001.

To evaluate the function of TCP1 in drug resistance of AML cells in vivo, AML xenografts in athymic nude mice were employed as a model system. TCP1 overexpression in HL60 cells promoted the growth of AML xenografts and impeded ADM-mediated growth inhibition in vivo (Fig. 6A–C). Immunofluorescence staining of tumor tissues demonstrated that TCP1 overexpression inhibited the LC3 expression (Fig. 6D). Western blotting showcased that TCP1 overexpression activated AKT and mTOR, significantly attenuated the ADM-induced increase in LC3-II, LC3-II/LC3-I ratio, and cleaved PARP levels, and decreased in p62 level, which was consistent with in vitro results (Fig. 6E). These observations indicated that TCP1 overexpression activated AKT/mTOR and inhibited ADM-induced autophagy and apoptosis, which contributed to the chemoresistance of HL60 cells to ADM treatment in vivo.

## Discussion

Many tumors have TCP1 expression and TCP1 has been shown to be involved in drug resistance in breast cancer and ovarian cancer [10, 11]. In present study, we discovered that TCP1 was upregulated in relapsed patients and drug-resistant HL60/A and K562/A cells. High TCP1 expression was associated with low CR rate and poor overall survival. The TCP1 downregulation can improve their sensitivity to ADM, whereas TCP1 overexpression confers resistance to the therapeutic effects of ADM. Thus, these results suggest that high TCP1 level was associated with drug resistance in AML.

Drug resistance is a major obstacle in the AML treatment. Many mechanisms of drug resistance including autophagy are well established. Both prosurvival and pro-death roles of autophagy have been proposed in AML cells in response to genetic background and therapeutic stress, which are dependent on the cellular context and duration or degree of stress stimuli. Moreover, accumulating evidence has revealed a correlative relationship between overcoming chemoresistance and activated autophagic activity in AML cells [13–18, 29–34]. In the current study, we demonstrated for the first time that TCP1 inhibition in HL60/A and K562/A cells directly increases autophagy, whereas TCP1 overexpression in HL60 cells represses autophagic flux. Evidences suggested a crosstalk between autophagic and apoptotic pathways [35], and recent studies showed that autophagy played a pro-death role in AML cells treated with chemotherapeutic agents by enhancing autophagy-mediated apoptosis instead of autophagic cell death [7, 31–34]. Our study had similar results, showing that knocking down TCP1 in HL60/A and K562/A cells significantly increased ADM-induced apoptosis, and this effect was attenuated by inhibiting autophagy. In contrast, overexpressing TCP1 in HL60 cells significantly decreased ADM-induced apoptosis and this effect was partially reversed by RAPA, which activated autophagy. Taken together, our findings reveal a novel mechanism by which TCP1 protects AML cells against apoptosis resulting from ADM treatment by partly inhibiting autophagy.

Several autophagic pathways that are involved have been identified [36] and the mTOR signal is a key signal in the autophagy initiation [24, 25]. Studies have shown that the AKT/mTOR axis strongly contributes to drug resistance [26] and inhibiting AKT/mTOR signaling can promote chemosensitivity in AML cell lines [37, 38]. In addition, previous study showcased that CCT could regulate mTOR signal and knocking down some of the subunits of CCT (CCT2 and CCT5) can inhibit the mTOR signal [5]. TCP1 is one of the CCT subunits, which may have similar functions to CCT2 or CCT5. Our data showcased that phosphorylation levels of AKT and mTOR are positively correlated with TCP1 expression. The effects of TCP1 overexpression in HL60 cells were significantly antagonized by LY294002 and RAPA, which are AKT/mTOR inhibitors. We observed physical interactions between TCP1 and AKT, as well as between TCP1 and mTOR by co-immunoprecipitation assays. However, further in-depth studies are necessary to elucidate the molecular mechanisms underlying the activation of AKT or mTOR by TCP1.

In one word, the present study showcased that TCP1 protected AML cells from ADM-induced apoptosis and increased drug resistance, which were mediated in part by the autophagy inhibition. Mechanistically, the negative regulation of autophagy and apoptosis by TCP1 is mediated by AKT/mTOR signaling. These new insights contribute to the understanding of the mechanism by which TCP1 enhances the resistance of AML cells to chemotherapy and identifies TCP1 as a potential therapeutic target for treating drug-resistant AML.

## Supplementary information

Supplementary figure legend

supplementary Fig1

supplementary Fig2

supplementary Fig3

## Data Availability

The data sets used and/or analyzed during the current study are available upon reasonable requests to corresponding author.
