# Supplementary material for: TCP1 increases drug resistance in acute myeloid leukemia by suppressing autophagy via activating AKT/mTOR signaling
Source: Cell Death Dis. 2021 Nov 8;12(11):1058. doi: 10.1038/s41419-021-04336-w (PMC8575913; doi:10.1038/s41419-021-04336-w)
Supplement: Supplementary file 1 — Supplementary figure legend [file 41419_2021_4336_MOESM1_ESM.doc]

**Supplementary Figure Legends**

**Supplementary Fig. 1.**

**Knockdown of TCP1 in the HL60 cells enhance autophagy.**

**(A)** Cells were stained with anti-LC3A/B antibody and DAPI to detect the endogenous LC3 puncta and nuclei, respectively. Immunofluorescence was performed using confocal microscope. Scale bar is 25 μm. The quantitative analyses of the number of fluorescent puncta are shown. **(B)** Western blot analysis of LC3-II, LC3-I, and TCP1 expression levels in the presence or absence of 10 μM HCQ in cells. Data are expressed as mean ± SEM; **P* < 0.05, ***P* < 0.01, ****P* < 0.001.

**Supplementary Fig. 2.**

**Rapamycin activates autophagy and promote** **ADM-induced apoptosis in AML cells.**

**(A)** HL60/A and K562/A cellS were treated with RAPA (1 μM) for 48 h,then the cells were stained with anti-LC3A/B antibody and DAPI to detect the endogenous LC3 puncta and nuclei, respectively, by immunofluorescence (Scale bar = 25 μm) and the quantitative analyses of the number of fluorescent puncta are shown. HL60/A and K562/A cells were treated with or without RAPA (1 μM) for 2 h and then combined with or without ADM for 48 h. **(B)** Apoptotic cells were assessed using flow cytometry, and the percentage of early and late apoptotic cells were quantified. **(C)**The levels of TCP1, mTOR, p-mTOR, autophagy- and apoptosis-related molecules were detected using western blotting. Data are denoted by mean ± SEM; **P* < 0.05, ***P* < 0.01, ****P* < 0.001.

**Supplementary Fig. 3.**

**Inhibition of autophagy by TCP1 contributes to inhibit ADM-induced apoptosis in AML cells.**

Autophagy- and apoptosis - related protein were detected using western blotting after the cells were treated as follows:**(A,B)**HL60/A -shTCP1 and K562/A-shTCP1 cells were treated with or without 4 μM ADM for 48h after incubation with 10 μM HCQ for 2 h. **(C)** HL60/ADM-shTCP1 cells were transfected with ATG7 shRNA or negative control (NC) for 72 h and then treated with ADM (4 μM) for 48 h,**(D)** HL60/ TCP1 cells were treated with or without 0.5 μM ADM after incubation with rapamycin (RAPA, 1 μM) for 2 h.
