# Supplementary figures and images for: TCP1 increases drug resistance in acute myeloid leukemia by suppressing autophagy via activating AKT/mTOR signaling
Source: Cell Death Dis. 2021 Nov 8;12(11):1058. doi: 10.1038/s41419-021-04336-w (PMC8575913; doi:10.1038/s41419-021-04336-w)

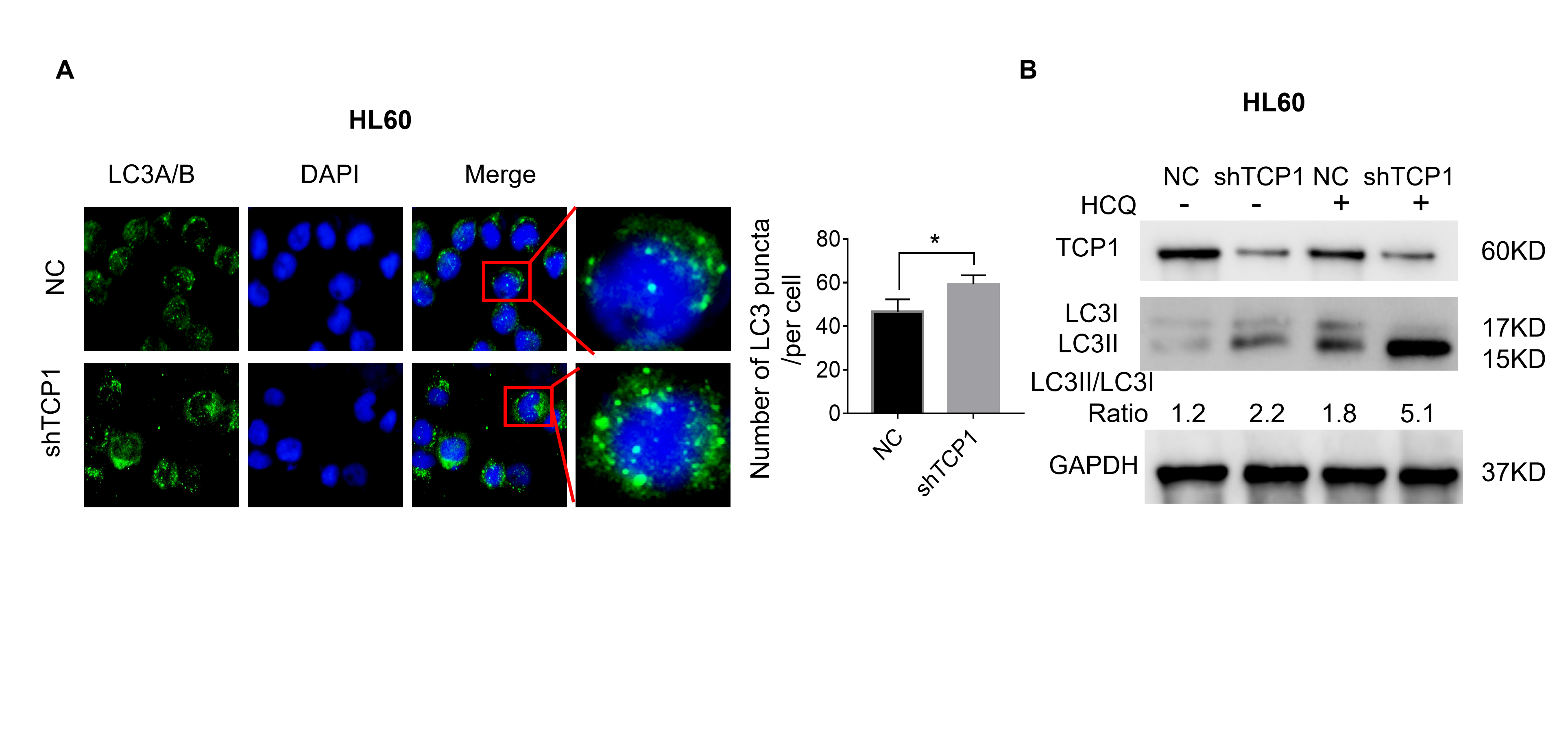

Supplement: Supplementary file 2 — supplementary Fig1 [file 41419_2021_4336_MOESM2_ESM.tif]

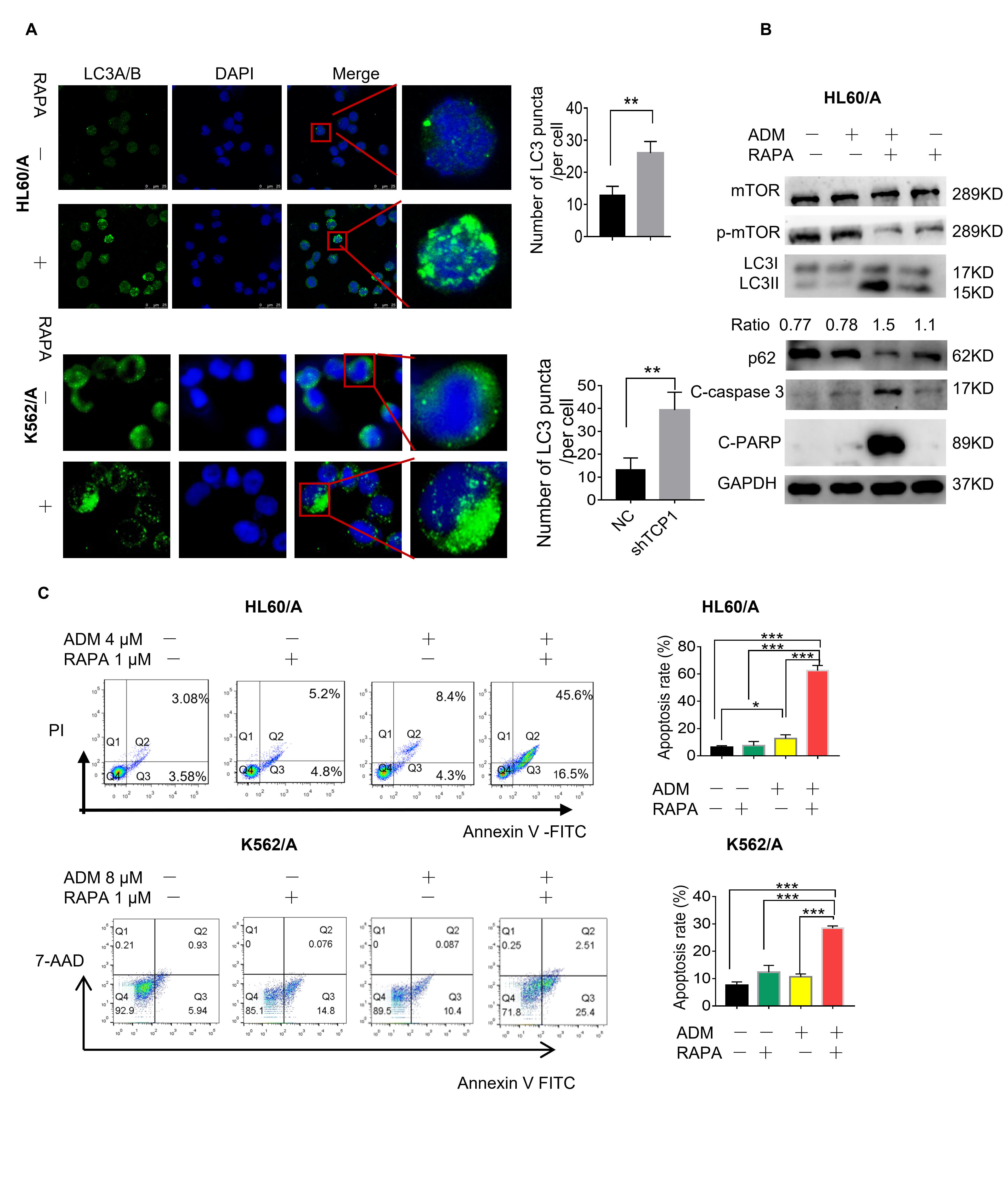

Supplement: Supplementary file 3 — supplementary Fig2 [file 41419_2021_4336_MOESM3_ESM.tif]

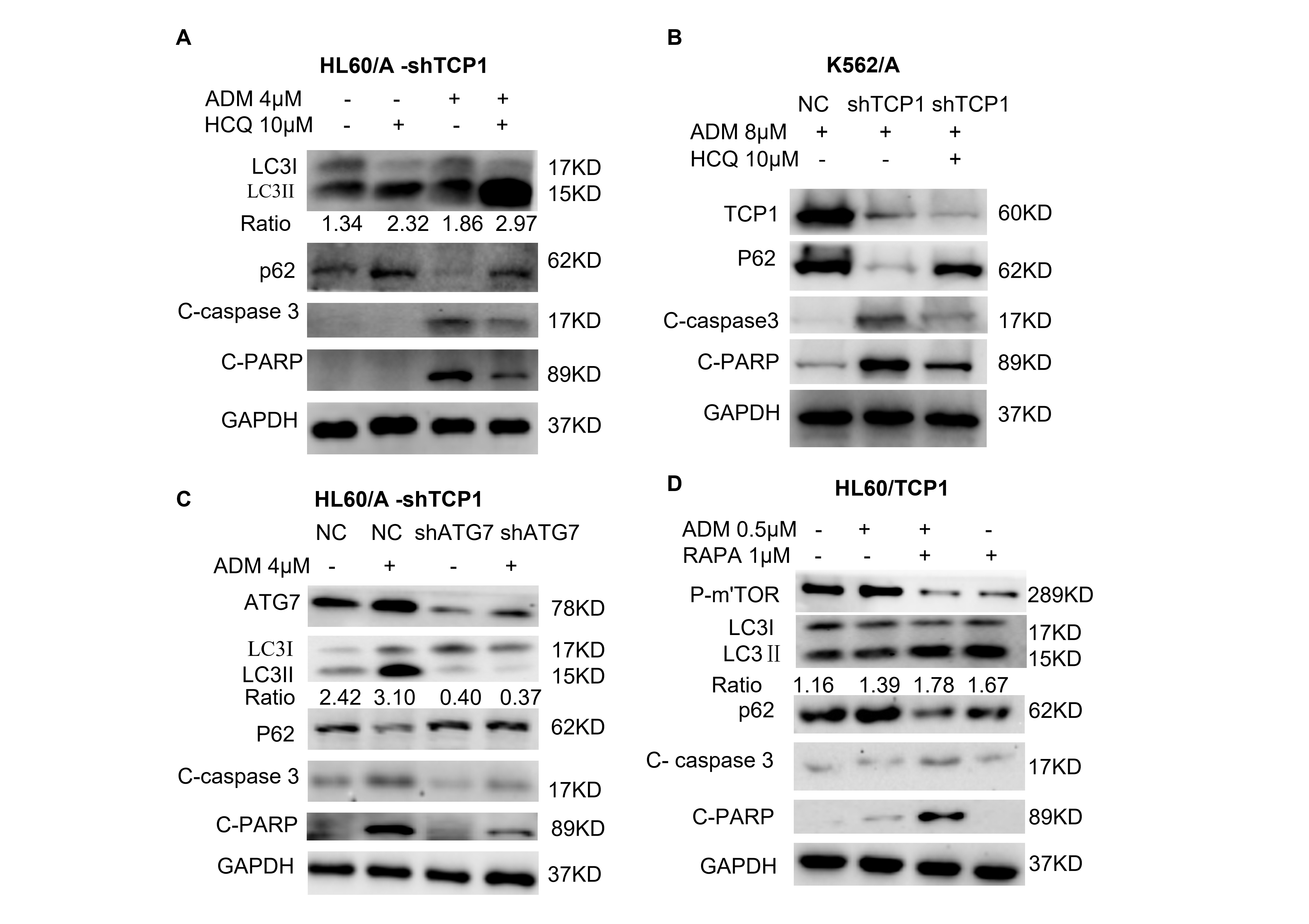

Supplement: Supplementary file 4 — supplementary Fig3 [file 41419_2021_4336_MOESM4_ESM.tif]
